# Concomitant occurrence of chronic *Schistosoma mansoni* infection and chronic colitis restore immune imbalance and dysbiosis leading to protection against intestinal colitis and schistosome egg-induced intestinal fibrosis

**DOI:** 10.1590/0074-02760240045

**Published:** 2025-05-02

**Authors:** You-Ren Lin, Long Yin Lam, Chun-Ming Chang, Ho Yin Pekkle Lam

**Affiliations:** 1 Tzu Chi University, School of Medicine, Master Program in Biomedical Sciences, Hualien, Taiwan; 2 The Hong Kong Polytechnic University, Department of Applied Biology and Chemical Technology, State Key Laboratory of Chemical Biology and Drug Discovery, Kowloon, Hong Kong SAR, China; 3 Buddhist Tzu Chi Medical Foundation, Hualien Tzu Chi Hospital, Department of General Surgery, Hualien, Taiwan; 4 Tzu Chi University, Institute of Medical Sciences, Hualien, Taiwan; 5 Tzu Chi University, School of Medicine, Department of Biochemistry, Hualien, Taiwan

**Keywords:** Schistosoma mansoni, dextran sodium sulphate, fibrosis, inflammatory bowel disease, dysbiosis

## Abstract

**BACKGROUND:**

Schistosomiasis is one of the most devastating tropical diseases in developing countries and is usually misdiagnosed with colitis because the prevalence of co-occurrence of both diseases is high. Previously, infection of *Schistosoma japonicum* cercariae has been shown to provide immediate protection against dextran sodium sulphate (DSS)-induced acute colitis in mice models. Studies using synthesised peptides or soluble proteins from parasites also revealed similar protection against colitis. However, most of these studies were done within a short timeframe, which cannot completely represent the actual situation where natural infection of *Schistosoma* or colitis is usually chronic.

**OBJECTIVES:**

This study aims to investigate how chronic schistosomiasis affects chronic intestinal inflammation.

**METHODS:**

Mice were infected with *Schistosoma mansoni* and induced simultaneously with chronic colitis. The symptoms and severity of intestinal inflammation and fibrosis were investigated by disease activity index, histology, enzyme-linked immunosorbent assay (ELISA), and quantitative polymerase chain reaction (qPCR). Furthermore, immune analysis by ELISA and qPCR and microbiome analysis by 16S rDNA sequencing were done to investigate the underlying mechanism.

**FINDINGS:**

Concomitant occurrence of chronic schistosomiasis and chronic colitis significantly alleviated colitis symptoms, lessened intestinal inflammation, and reduced egg-induced fibrosis. Further analysis revealed an alternation of the intestinal immunity and gut microbiome community in mice with both diseases, which could be the potential reason for this outcome.

**MAIN CONCLUSIONS:**

Our results represent a mechanism of how schistosomiasis and chronic intestinal inflammation affect each other.

Inflammatory bowel disease (IBD), affecting millions of people worldwide, runs the spectrum from ulcerative colitis to Crohn’s disease. Although the pathogenesis of IBD is still unclear, it is characterised by a dysregulated immune response, leading to recurrent intestinal inflammation.^1^ In most developing countries or tropical countries, IBD is sometimes misdiagnosed by parasitic diseases such as amebiasis or schistosomiasis as the prevalence of both IBD and parasite infection is high in these countries; in addition, they share overlapping endoscopic and radiological findings.^2^ ^,^ ^3^

Interestingly, epidemiological evidence has suggested that a low incidence of parasitic diseases may negatively correlate to the incidence of autoimmune diseases.^4^ The hypothesis, known as the hygiene hypothesis, suggested that certain parasites such as *Schistosoma* and *Necator americanus* may harness an immune-modulatory effect on the infected host, ameliorating or preventing autoimmune diseases.^5^ ^,^ ^6^ Pre-clinical and clinical studies have provided evidence that certain proteins found on *Schistosoma mansoni*,^7^ *Ancylostoma ceylanicum*,^8^ *N. americanus*,^9^ and *Trichuris suis* ^10^ ^,^ ^11^ can modulate the host immunity to alleviate colitis symptoms.

Of all parasites, *Schistosoma* has been known as a strong immune-modulatory parasite.^12^ Throughout the life cycle, schistosome employs many immune evasion strategies to facilitate their survival in the host. Once cercariae penetrate the human skin, they release numerous antigens such as Sm16, which suppress antigen-presentation.^13^ Mature worms in mesenteric blood vessels then follows by secretion of immunosuppressive molecules such as *S. mansoni* Kunitz type protease inhibitor (SmKI) which inhibits neutrophil elastase^14^ or *S. japonicum* thioredoxin peroxidase-1 (SjTpx) which inhibits major histocompatibility complex (MHC)-II expression on macrophages, affecting antigen presentation.^15^ Together, these molecules allow the worm to evade host immunity and provide the worm with a favourable immune environment. Finally, the eggs laid by the adults get trapped in different tissues. Antigens released from the egg recruit a large number of infiltrating immune cells, leading to a polarised Th2 immunity, inflammatory granulomatous reaction, and, consequently, fibrosis.^16^

Previously, cercariae of *S. japonicum* infection has been shown to provide immediate protection against dextran sodium sulphate (DSS)-induced colitis in mice model, by which cercariae suppressed splenic interferon (IFN)-γ and increased splenic IL-10 and regulatory T (Treg) cells.^17^ A similar study using a synthesised peptide from the HSP60 protein of *S. japonicum* revealed similar protection against acute and chronic DSS-induced colitis.^18^ However, most of these studies were done within a short timeframe of only seven days,^17^ ^,^ ^18^ ^,^ ^19^ which cannot completely represent the true situation where natural infection of *Schistosoma* or IBD is usually chronic. Here, we provide experimental evidence assessing the potential modulatory involvement of chronic schistosomiasis in chronic colitis.

## MATERIALS AND METHODS

*Animals and parasites* - Animal experiments were approved by the Institutional Animal Care and Use Committees (IACUC) of Tzu Chi University (No. 112050 and 113043). Male BALB/cByJNarl (BALB/c) mice were provided by the National Laboratory Animal Centre (NLAC), NARLabs, Taiwan. All mice were housed under a 23ºC ± 1ºC and a 12-h light/dark cycle condition with 40-60% humidity. Food and water were available *ad libitum*.

Puerto Rico strain of *S. mansoni* was provided by the Biomedical Research Institute, Rockville, MD, USA. The freshwater snail *Biomphalaria glabrata* was used as an intermediate host and male BALB/c mice were used as the final host.

**Fig. 1:**
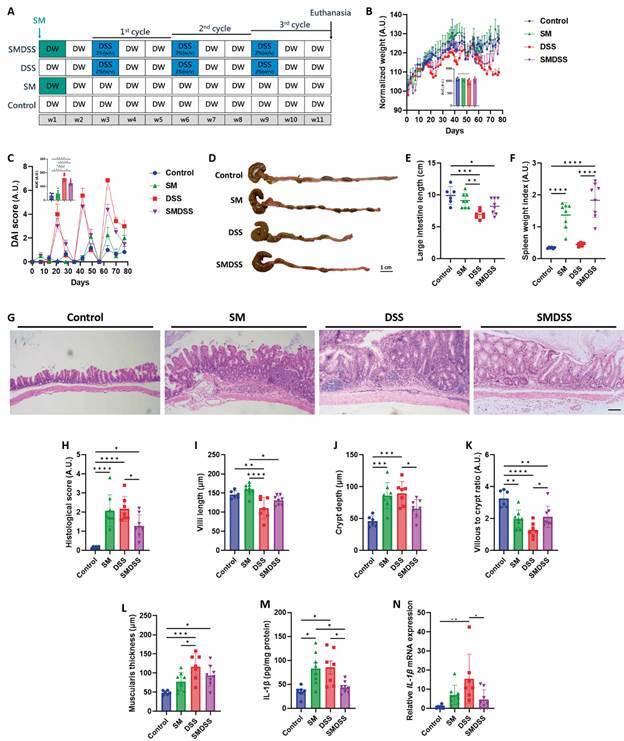
concurrent schistosomiasis and dextran sodium sulphate (DSS)-induced colitis resolve symptoms and intestinal inflammation. (A) Experimental scheme. Mice were infected with 80 ± 10 *Schistosoma mansoni* cercariae followed by three cycles of DSS treatment to induce chronic colitis. (B) Change of mice body weight and their area under curve (AUC) chart. (C) Disease activity index (DAI) of the mice and a corresponding AUC chart. (D) Representative image of the colon in each group of mice. Scale bar represents 1 cm. (E) Length of the colon. (F) Spleen weight index, calculated by the (spleen weight divided by the body weight) ×100. (G) Representative H&E-stained histological image of the colon. Scale bar represents 100 μm. (H) Histological score of the intestine. (I-L) Measurement of villi length (I), crypt depth (J), villi-to-crypt ratio (K), and muscularis thickness (L). (M) Cytokine level of IL-1β in the colon. The concentration was normalised to the total protein concentration. (N) mRNA expression level of IL-1β in the colon. The expression was normalised to the housekeeping gene glyceraldehyde-3-phosphate dehydrogenase (GAPDH). n = 6 mice in control group, n = 8 mice in *S. mansoni*-infected (SM) and *S. mansoni*-infected and DSS-treated (SMDSS) group, and n = 7 mice in DSS group. Data are presented as mean ± standard deviation (SD). ^*^p < 0.05; ^**^p < 0.01; ^***^p < 0.001; and ^****^p < 0.0001. Significance determined by one-way analysis of variance (ANOVA).

*Animal treatment* - Male mice were six weeks of age at the beginning of the experiments. Mice were divided randomly into control (n = 6), *S. mansoni*-infected (SM) (n = 8), DSS (n = 7), and *S. mansoni*-infected and DSS-treated (SMDSS) (n = 8) groups. Mice from the SM and SMDSS groups were subcutaneously infected with 80 ± 10 *S. mansoni* cercariae, whereas the mice from the other groups were treated with sterile water. Two weeks post-infection, mice from the DSS and SMDSS group were given 2% (w/v) DSS (MP Biochemical, Santa Ana, CA, United States) in drinking water for seven days, followed by two consecutive weeks of normal drinking water. The DSS cycle was repeated three times. All the mice were euthanised one day after the third cycle of DSS treatment (11 weeks post-cercariae infection; Fig. 1A).

*Disease activity index (DAI)* - The DAI was evaluated by three criteria: percentage of weight loss, presence of rectal bleeding, and stool consistency every week. Each criterion was assigned a score from 0 to 4 [Supplementary data (Table I)], and the sum of the three scores is calculated as the DAI score.

*Tissue processing, staining, and histopathology* - Colon, spleen, and liver were fixed with 10% formalin, embedded in paraffin, and sectioned into thin slices for haematoxylin & eosin (H&E), Masson trichrome, and Periodic acid-Schiff (PAS) staining as previously described.^20^ Colonic sections were scored for epithelial damage, lamina propria inflammation, muscularis propria thickening, and fibrosis. Each criterion was assigned a score of 0, not observed; 1, mild; 2, moderate; 3, intensive. Measurements were also done on villous height, crypt depth, villi-to-crypt ratio, and muscularis thickness. Liver sections were scored for steatosis, inflammation, necrosis, and fibrosis; each criterion was assigned a score of 0, not observed; 1, mild; 2, moderate; 3, intensive. Splenic sections were scored for the enlargement of white pulp (0, not observed; 1, mild; 2, moderate; and 3, pronounced) and the presence of apoptosis, necrosis, pigments, and macrophages (for each: 0, absent and 1, present). At least ten random fields were examined and scored in each section. Experiments were independently scored by two researchers in a blinded fashion.

*RNA extraction, cDNA synthesis, and quantitative polymerase chain reaction (qRT-PCR)* - Total RNA was extracted by homogenising tissues in TRIzol reagent (Invitrogen; Thermo Fisher Scientific, Waltham, MA, USA). RNA was then purified using the standard chloroform extraction method. Five micrograms of total RNA were used to generate cDNA using a GScript First-Strand Synthesis Kit (GeneDireX, Inc, Taiwan). The qPCR reaction was performed by 2× qPCRBIO SyGreen Blue Mix Lo-ROX (PCR Biosystems, London, UK) using the Roche LightCycler 480 system. Amplification and detection were performed as follows: 45 cycles of denaturation at 95ºC for 15 s, 60ºC for 25 s, and extension at 72ºC for 20 s. The oligonucleotide primers used are shown in Supplementary data (Table II). Relative gene expression was calculated using the 2^−ΔΔCT^ method with glyceraldehyde-3-phosphate dehydrogenase (GAPDH) as the reference housekeeping gene. GAPDH was assured to be insensitive to the experimental conditions in this study and was identified by the algorithm NormFinder^21^ among a set of commonly used housekeeping genes [Supplementary data (Fig. 1)].

*Measurement of cytokine and ALT levels* - Levels of IL-1β, IL-2, IL-4, IL-5, IL-10, and IFN-γ in the sera or tissue homogenate were measured using a standard sandwich enzyme-linked immunosorbent assay (ELISA) kit (Cat#: 432604 for IL-1β; BioLegend, San Diego, CA, USA; Cat#: 88-7711-44 for IL-4, IL-10, IL-2, and IFN-γ; Cat#: 88-7054-22 for IL-5; Thermo Fisher Scientific). Protein concentrations of tissue homogenates were determined by the Bradford method using a Bio-Rad Protein Assay Dye (Bio-Rad Laboratories, Hercules, CA, USA). Serum alanine transaminase (ALT) levels were measured by an ALT liquicolour kit (HUMAN Diagnostics Worldwide, Wiesbaden, Germany).

*Quantification of schistosome eggs* - Faecal eggs were counted from a weighted stool using the Kato-Katz technique.^22^ Intestinal or hepatic eggs were counted on weighted tissue fractions after digesting with 4% KOH for 4 h.^23^

*DNA extraction, 16S rDNA sequencing, and analysis* - Total DNA from colonic samples was extracted with a tissue genomic DNA spin kit (Bioman Scientific, New Taipei City, Taiwan) following the manufacturer’s instructions. DNA purity and concentration were monitored by a NanoDrop 2000 spectrophotometer and Qubit dsDNA HS assay kit (Invitrogen). PCR amplification was performed spanning the V3 and V4 regions using the forward primers 5’-CCTACGGRRBGCASCAGKVRVGAAT-3’ and reverse primers 5’-GGACTACNVGGGTWTCTAATCC-3’ of the 16S rDNA gene and subsequently sequenced using 250 bp paired-end sequencing (Illumina MiSeq, Illumina, San Diego, USA).

Bcl2fastq (v2.20) was used to analyse the original image data. Overlapping paired-end FASTQ files were matched and processed in QIIME version 1.9.1. Adapter sequences were removed by Cutadapt version 1.9.1 before the resulting sequence data were aligned to the database. Operational taxonomic unit (OTU) clustering was done using QIIME version 1.9.1 and VSEARCH version 1.9.6, with sequence similarity set to 97%. The Ribosomal Database Project (RDP) classifier, Bayesian algorithm, was used to classify the OTU representative sequences. Taxonomic annotation was assigned based on the Silva_138 16S rRNA database (http://www.arbsilva.de/), ITS database (https://unite.ut.ee/), and NCBI database (https://www.ncbi.nlm.nih.gov/). Alpha diversity and beta diversity were calculated by QIIME version 1.9.1. Ordination plots were calculated from the Brary-Curtis distance matrix using principal coordinate analysis (PCoA).

All the raw sequencing data were deposited in the Sequence Read Archive (SRA) public database under the bio-project PRJNA1128329 and accession number SAMN42043612-38.

*Statistical analysis* - Data are expressed as mean ± standard deviation (SD) unless stated otherwise. One-way analysis of variance (ANOVA) was used with Tukey’s honest significant difference test to estimate differences between groups. An unpaired t-test was used to analyse the difference between the two groups. Kruskal-Wallis test was used in alpha-diversity analysis to analyse the difference between groups. PCoA analysis was performed and plotted based on the Brary-Curtis distance matrix. Multivariate analysis of variance (MANOVA) was used to analyse the relevance of each grouping factor in beta-diversity analysis. All analyses were done by R software version 3.3.1 or GraphPad Prism software version 9.4.1. p < 0.05 were considered significant (*p < 0.05; **p < 0.01; ***p < 0.001; ****p < 0.0001).

## RESULTS

**Fig. 2:**
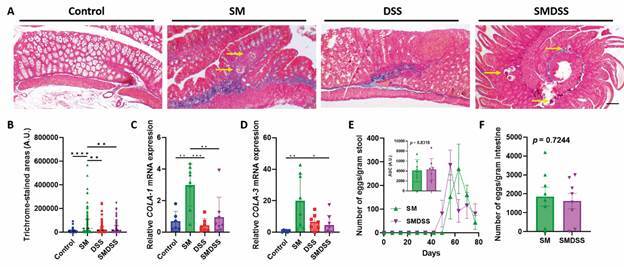
concurrent schistosomiasis and dextran sodium sulphate (DSS)-induced colitis reduce egg-induced intestinal fibrosis. (A) Representative Masson trichrome-stained histological image of the colon. Blue colour represents stained collagen. Yellow arrows point to schistosome eggs. The scale bar represents 100 μm. (B) Quantification of all trichrome-stained areas in each group of mice. (C-D) Colonic mRNA expression level of COLA-1 (D) and COLA-3 (E). The expression was normalised to the housekeeping gene glyceraldehyde-3-phosphate dehydrogenase (GAPDH). (F) Number of eggs counted in the stool and their corresponding area under curve (AUC). (G) Number of eggs counted per gram intestine. n = 6 mice in control group, n = 8 mice in *Schistosoma mansoni*-infected (SM) and *S. mansoni*-infected and DSS-treated (SMDSS) group, and n = 7 mice in DSS group. Data are presented as mean ± standard deviation (SD). ^*^p < 0.05; ^**^p < 0.01; ^***^p < 0.001; and ^****^p < 0.0001. (B, D, and E) Significance determined by one-way analysis of variance (ANOVA). (F and G) Significance determined by t-test.

*Co-occurrence of schistosomiasis and chronic colitis alleviates symptoms of colitis and intestinal histopathology from both diseases* - To assess the effect of chronic schistosomiasis on colitis, we employed the use of *S. mansoni*-infected mice (SM). First, the mice were allowed to develop chronic schistosomiasis by cercariae infection. During the infection, mice were fed three cycles of DSS to induce chronic colitis (Fig. 1A). Symptoms of the mice were assessed by measuring the body weight and disease activity index (DAI). While the body weight of SM mice was similar to that of control mice, body weight of DSS mice significantly decreased after the third cycle of DSS treatment. On the other hand, the body weight of DSS mice was improved by SM infection (SMDSS mice, Fig. 1B). Similarly, DSS mice had a significantly higher DAI score compared to both control and SM mice, which was suppressed by SM infection (SMDSS mice; Fig. 1C). Although SM infection induced intestinal manifestation, colon length of SM mice was only slightly decreased compared to control mice. Sole DSS treatment induced a more significant decrease in colon length, which was somewhat improved by SM infection (Fig. 1D-E). Mice infected with SM also showed splenomegaly, but the effect was more pronounced when SM mice were treated with DSS (Fig. 1F). Histological analysis revealed egg entrapment and inflammation occupying mucosa and submucosa in SM mice, whereas in DSS mice there was continuous inflammation with cryptitis and distortion of intestinal structure. In SMDSS mice, the histology was significantly improved, with lesser inflammatory infiltration and structural distortion compared to SM or DSS mice [Fig. 1G-H, Supplementary data (Fig. 2)]. By measuring the villous length and crypt depth, we found that the villous-to-crypt ratio was decreased in both SM and DSS mice compared to normal mice. This intestinal damage was protected by co-occurrence of SM and colitis (Fig. 1I-K). A similar finding was found by measuring muscularis thickness (Fig. 1L). Finally, attempts to analyse the extent of intestinal inflammation revealed a significant increase of intestinal interleukin (IL)-1β level in SM and DSS mice, and the level was reduced in SMDSS mice (Fig. 1M). Similar results were observed in the gene expression, showing a decreased *IL-1β* expression in SMDSS mice compared to DSS mice (Fig. 1N). These results identified that co-occurrence of chronic schistosomiasis and colitis can alleviate colitis symptoms and intestinal inflammation.

*Co-occurrence of schistosomiasis and chronic colitis reduces egg-induced intestinal fibrosis* - We next investigated intestinal fibrosis because egg entrapment usually leads to granulomatous inflammation, consequently leading to fibrogenesis.^12^ Compared to SM mice, SMDSS mice showed a significant reduction in intestinal fibrosis [Fig. 1G-H, Supplementary data (Fig. 2)]. Masson trichrome staining was next performed to investigate the degree of fibrosis. As suggested, an increased collagen deposit was found surrounding the schistosome eggs in SM mice. Collagen depositions were also noted in some parts of the intestine of DSS mice. However, in SMDSS mice, only a minimal amount of collagen deposits was found surrounding the eggs (Fig. 2A). As collagen deposition was significantly lowered in SMDSS mice compared to SM mice (Fig. 2B), fibrotic markers were investigated in the intestine. In accordance with the histology, the intestine of SMDSS mice showed a decreased *collagen (COLA)-1* and *COLA-3* expression (Fig. 2C-D) compared to that of SM mice, providing evidence that co-occurrence of schistosomiasis and colitis resolves egg-induced fibrosis.

Because the severity of fibrosis positively correlates with the number of eggs, we investigated whether the reduction of intestinal fibrosis in SMDSS mice was related to their lodged eggs. After counting the number of eggs in the stool (Fig. 2E) and intestine (Fig. 2F), we revealed no differences in the number of eggs between SM mice and SMDSS mice. We also found no difference between the viability of tissue-entrapped eggs, as intact egg shells, but not ruptured eggs, were seen in all of the tissue sections. All of these results suggested that the observed improvement of fibrosis was not caused by the reduction of the number of eggs or their viability.

**Fig. 3:**
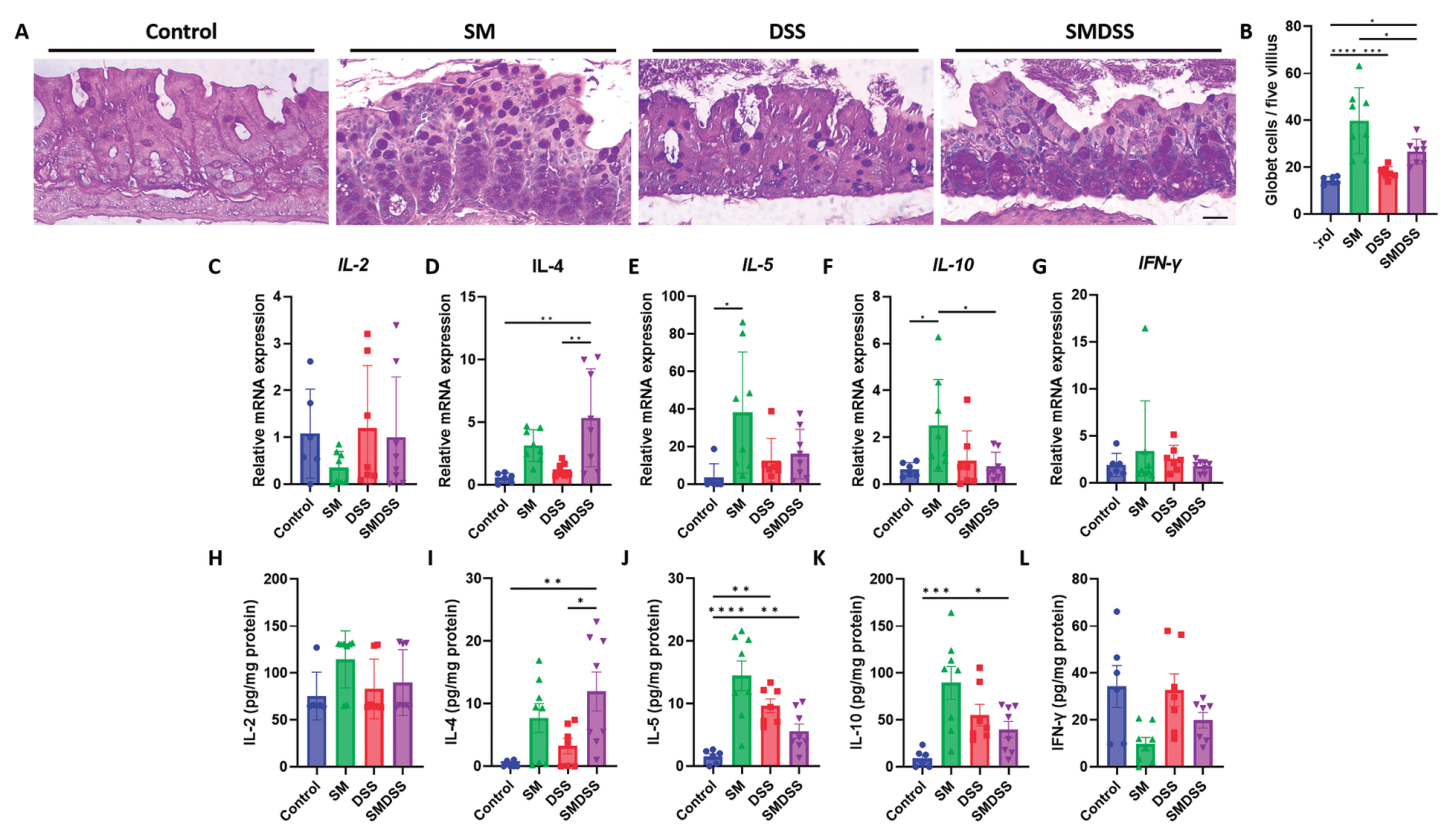
concurrent schistosomiasis and dextran sodium sulphate (DSS)-induced colitis altered intestinal immunity. (A) Representative Periodic acid-Schiff (PAS)-stained histological image of the colon. Scale bar represents 100 μm. (B) Quantification of goblet cells per five villous. (C-G) mRNA expression level of IL-2 (C), IL-4 (D), IL-5 (E), IL-10 (F), and IFN-γ (G) in the colon. The expression was normalised to the housekeeping gene glyceraldehyde-3-phosphate dehydrogenase (GAPDH). (H-L) Cytokine level of IL-2 (H), IL-4 (I), IL-5 (J), IL-10 (K), and IFN-γ (L) in the colon. The concentration was normalised to the total protein concentration. n = 6 mice in control group, n = 8 mice in *Schistosoma mansoni*-infected (SM) and *S. mansoni*-infected and DSS-treated (SMDSS) group, and n = 7 mice in DSS group. Data are presented as mean ± standard deviation (SD). ^*^p < 0.05; ^**^p < 0.01; ^***^p < 0.001; and ^****^p < 0.0001. (B, D, and E) Significance determined by one-way analysis of variance (ANOVA).

In our experimental model, although DSS was mainly known to induce intestinal inflammation, we further explored the pathology of the liver, as SM infection also led to liver fibrosis. As expected, SM infection resulted in significant liver necrosis, inflammation, and fibrosis, but the pathology had no other effect when combined with DSS use [Supplementary data (Fig. 3A-B)]. Similarly, liver weight [Supplementary data (Fig. 3C)] and serum ALT levels [Supplementary data (Fig. 3D)] are similar between SM and SMDSS mice. The number of hepatic eggs [Supplementary data (Fig. 3E)] is also similar between the two groups. These results suggested that the co-occurrence of schistosomiasis and colitis affects only intestinal pathology but not liver pathology.

*Co-occurrence of schistosomiasis and chronic colitis manipulates the immune response* - Immunity plays a major part in schistosomiasis and colitis; on that account, we further examined how intestinal immunity takes part in these diseases’ co-occurrence. First, mucin production from goblet cells was examined by PAS staining as it provides a frontline of intestinal defence. Our results revealed a higher number of intestinal PAS-stained cells in SM mice than in control or DSS mice; the number of cells, on the other hand, decreased in SMDSS mice compared to SM mice (Fig. 3A-B). We next investigate the immune response within the intestine. As evident from the qRT-PCR and ELISA results, we noted an increase in intestinal Th2 cytokines, including IL-4 (Fig. 3D, I), IL-5 (Fig. 3E, J), and IL-10 (Fig. 3F, K) in SM mice. On the other hand, DSS mice showed only an increase in intestinal IL-5 (Fig. 3J) and IL-10 (Fig. 3K). These results are in accordance with previous results.^24^ ^,^ ^25^ However, in SMDSS mice, IL-4 was further exacerbated, where IL-5 and IL-10 were reduced. As also noted, Th1 cytokines, including IL-2 (Fig. 3C, H) and IFN-γ (Fig. 3G, L), did not alter in any of these groups.

**Fig. 4:**
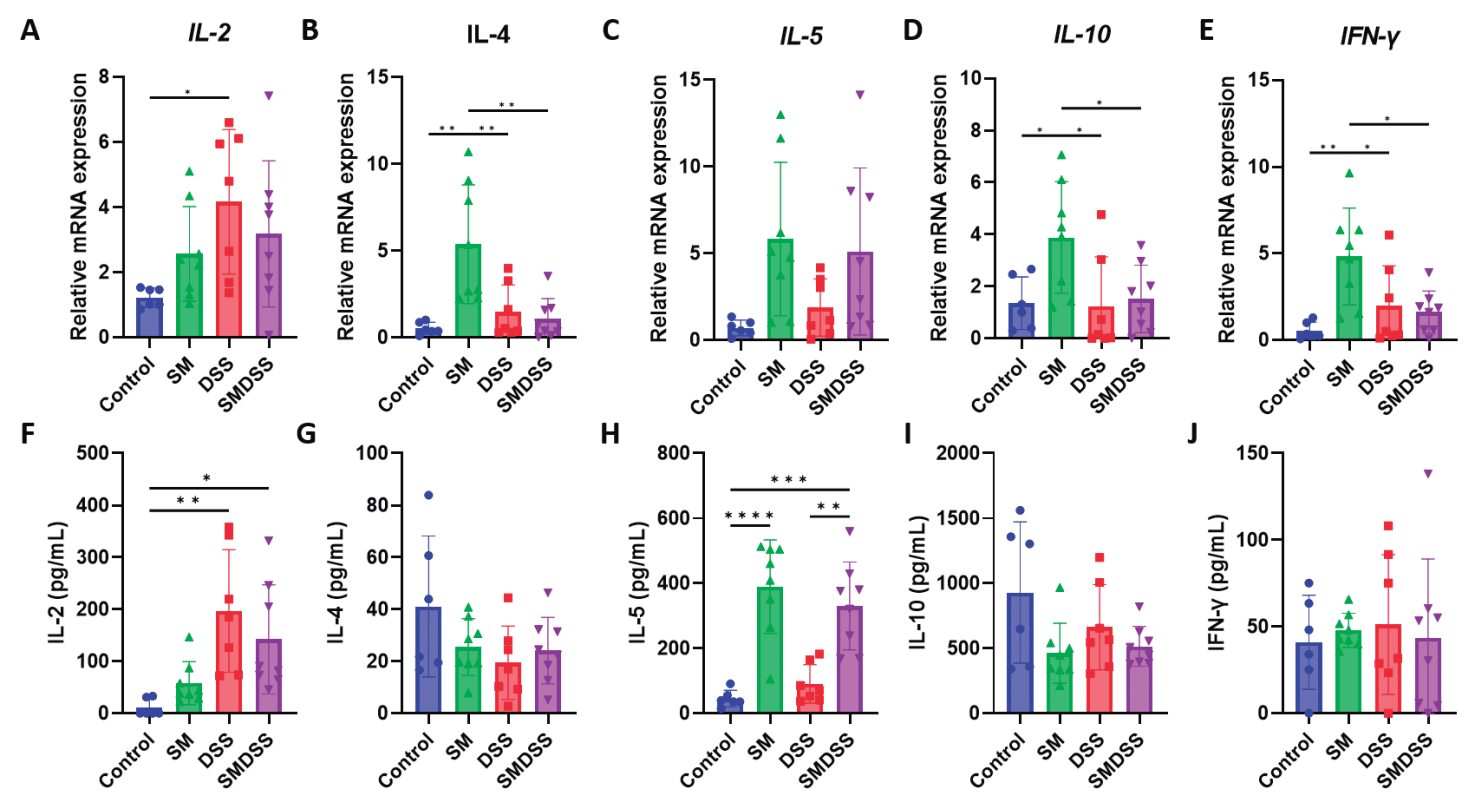
Immune response in the spleen and serum of mice. (A-E) mRNA expression level of IL-2 (A), IL-4 (B), IL-5 (C), IL-10 (D), and IFN-γ (E) in the spleen. The expression was normalised to the housekeeping gene glyceraldehyde-3-phosphate dehydrogenase (GAPDH). (F-J) Cytokine levels of IL-2 (F), IL-4 (G), IL-5 (H), IL-10 (I), and IFN-γ (J) in the serum. n = 6 mice in control group, n = 8 mice in *Schistosoma mansoni*-infected (SM) and *S. mansoni*-infected and DSS-treated (SMDSS) group, and n = 7 mice in dextran sodium sulphate (DSS) group. Data are presented as mean ± standard deviation (SD). ^*^p < 0.05; ^**^p < 0.01; ^***^p < 0.001; and ^****^p < 0.0001. (B, D, and E) Significance determined by one-way analysis of variance (ANOVA).

We also investigated cytokine expression in the spleen. We found that *IL-2*, *IL-4*, *IL-5*, *IL-10*, and *IFN-γ* were upregulated in SM mice (Fig. 4A-E); but only *IL-2* expression was upregulated in DSS mice compared to control mice. In SMDSS mice, the increased levels of *IL-4*, *IL-10*, and *IFN-γ* were reduced compared to SM mice. Serum cytokine levels were also measured to reflect the general state of the mice. While SM mice showed a significant increase of IL-5 (Fig. 4H), DSS mice showed only an increase in IL-2 levels (Fig. 4F). However, levels of these cytokines were not altered in SMDSS mice compared to SM or DSS mice.

The difference in the change of cytokine profile between the local immunity (intestine) and systematic immunity (spleen and serum) therefore suggests a distinctive role that helps to improve the pathology of both schistosomiasis and colitis.

**Fig. 5:**
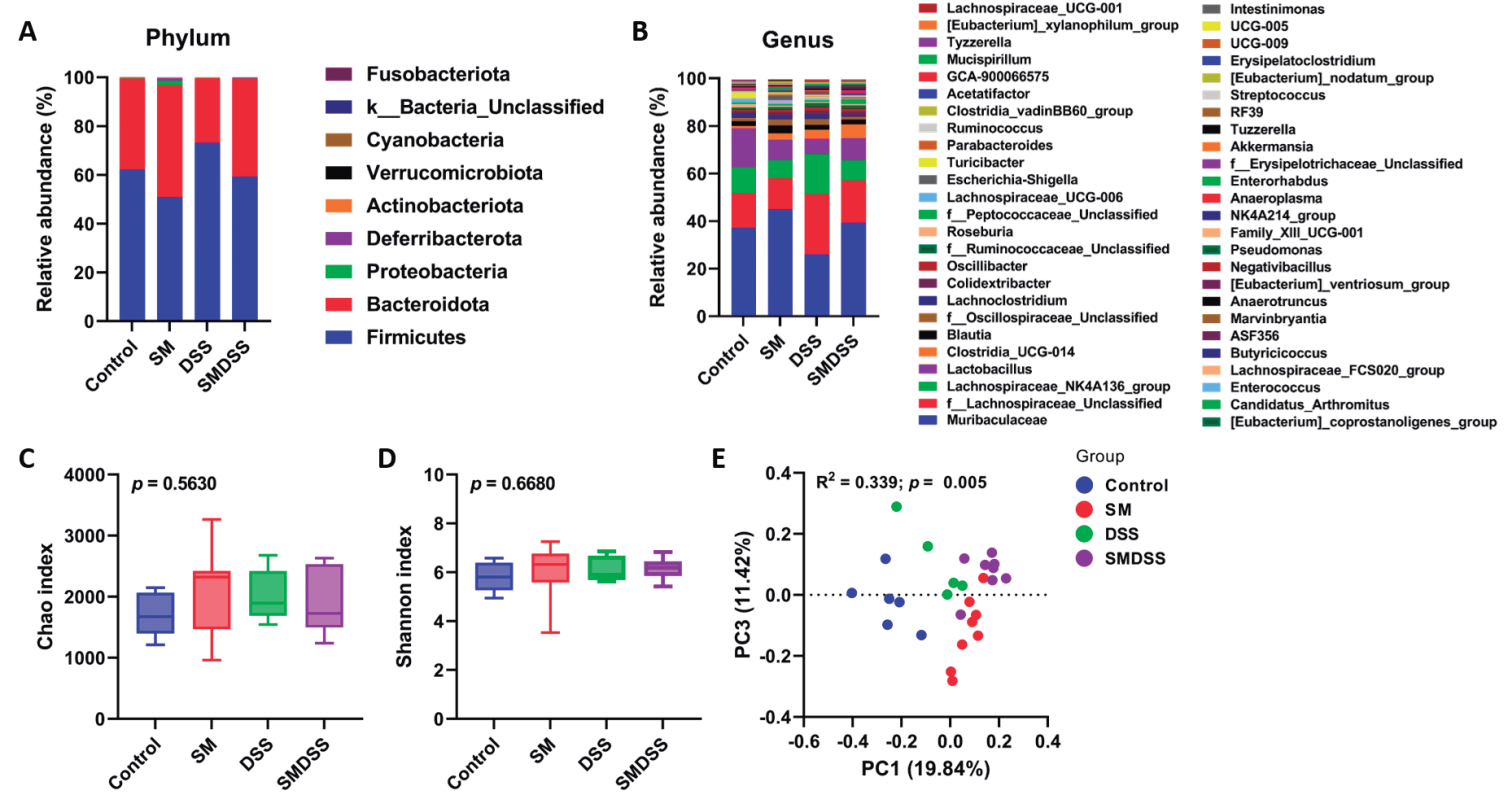
concurrent schistosomiasis and dextran sodium sulphate (DSS)-induced colitis altered the microbiome community but not microbiome diversity. (A-B) Averaged taxa summary of bacterial phyla (A) and genera (B) from each group of mice, obtained by 16S rDNA sequencing. (C-D) Alpha diversity index analysis including Chao index (C) and Shannon index (D). (E) PCoA plots, calculated using Bray-Curtis index. n = 6 mice in control group, n = 8 mice in *Schistosoma mansoni*-infected (SM) and *S. mansoni*-infected and DSS-treated (SMDSS) group, and n = 5 mice in DSS group. Box and whisker plot display the median at the central line, 25-75 percentile at the box, and 10-90 percentile at the whiskers. (C and D) Significance determined by one-way analysis of variance (ANOVA). (E) Significance according to multivariate analysis of variance (MANOVA).

*Co-occurrence of schistosomiasis and chronic colitis restores gut dysbiosis* - Finally, the intestinal microbiome was analysed by 16S rDNA sequencing to evaluate the role of gut microbiome in these diseases. However, no significant differences were observed in the bacterial phyla [Fig. 5A for averaged data and Supplementary data (Fig. 4A) for individual data] and genera among these groups [Fig. 5B for averaged data and Supplementary data (Fig. 4B) for individual data]. Although alpha diversity analysis revealed similar richness (Chao 1 index; Fig. 5C) and abundance (Shannon index; Fig. 5D) among different groups, beta diversity analysed by principal coordinate analysis (PCoA) revealed distinct distances, suggesting a diverse microbiome community between groups (Fig. 5E).

**Fig. 6:**
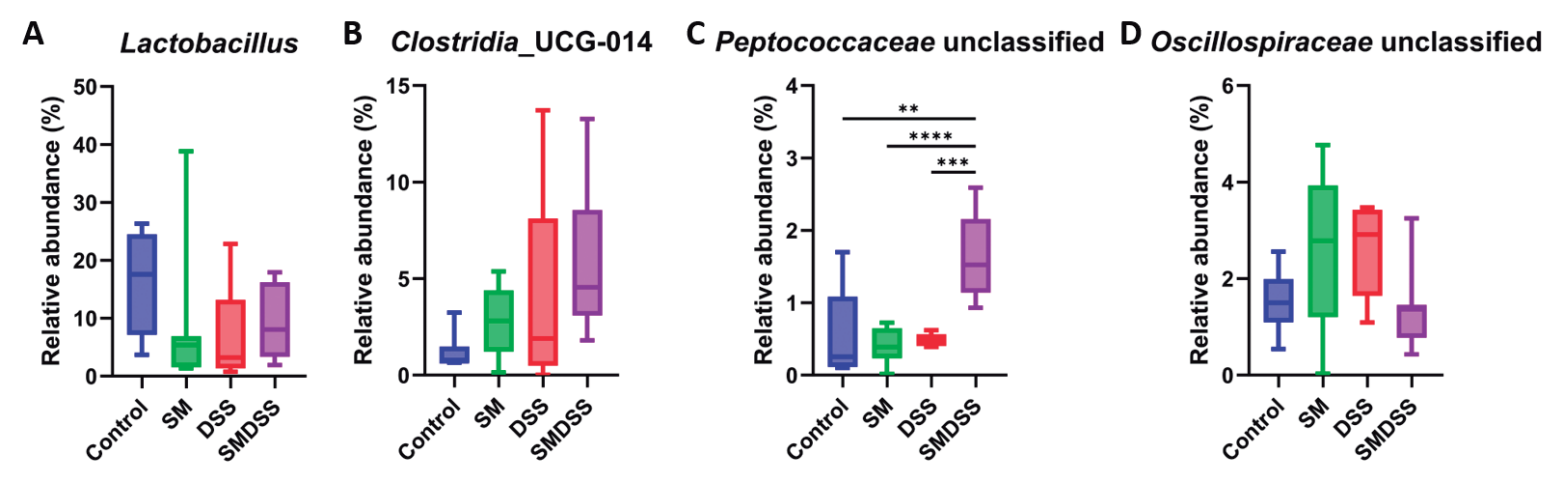
alternation of bacterial genera was observed in mice concurrent with schistosomiasis and dextran sodium sulphate (DSS)-induced colitis. Relative abundance of (A) *Lactobacillus*, (B) *Clostridia*_UCG-014, (C) *Peptococcaceae* family, and (D) *Oscillospiraceae* family, obtained by 16S rDNA sequencing. Box and whisker plot display the median at the central line, 25-75 percentile at the box, and 10-90 percentile at the whiskers. ^**^p < 0.01; ^***^p < 0.001; and ^****^p < 0.0001. Significance determined by one-way analysis of variance (ANOVA).

To further elucidate the role of microbiome in the co-occurrence of schistosomiasis and colitis, an LDA score was computed, showing a differential abundance of bacteria between groups [Supplementary data (Fig. 4C)]. Further analysis of specific bacteria showed that SM mice have a lower abundance of *Lactobacillus* (Fig. 6A), *Turicibacter* [Supplementary data (Fig. 5A)], and *Erysipelotrichaceae* family [Supplementary data (Fig. 5B)] and a higher abundance of *Clostridia*_UCG-014 (Fig. 6B) and *Oscillospiraceae* family (Fig. 6D). DSS mice showed a decrease abundance of *Lactobacillus* (Fig. 6A), *Turicibacter* [Supplementary data (Fig. 5A)], and *Erysipelotrichaceae* family [Supplementary data (Fig. 5B)], whereas an increased abundance was seen in *Oscillospiraceae* family (Fig. 6D). Finally, in SMDSS mice, abundance of *Lactobacillus* (Fig. 6A), *Clostridia*_UCG-014 (Fig. 6B), and *Peptococcaceae* family (Fig. 6C) increased, compared to SM or DSS mice. The abundance of the *Oscillospiraceae* family (Fig. 6D) was decreased compared to SM and DSS mice and returned to a similar abundance to control mice. However, changes in abundance were not seen in *Turicibacter* [Supplementary data (Fig. 5A)] and *Erysipelotrichaceae* family [Supplementary data (Fig. 5B)] in SMDSS mice. Therefore, mice with chronic schistosomiasis and colitis may have a partially restored gut microbiome compared to mice with only schistosomiasis or colitis.

## DISCUSSION

Currently, parasite infection has been shown to strongly affect other autoimmune diseases. In animal models, *S. mansoni* or *Fasciola hepatica* infection delayed the onset and symptoms of mice with experimental autoimmune encephalomyelitis.^26^ ^,^ ^27^ Parasite infection in multiple sclerosis patients lowered their disease exacerbations and radiological pathology,^28^ where the application of anti-parasitic drugs significantly worsened clinical responses.^29^ In a previous clinical study, infection of *T. suis* in patients with Crohn’s disease and ulcerative colitis resulted in remission of intestinal disease.^10^ In this study, we identified that concomitant occurrence of chronic schistosomiasi*s* and experimental chronic colitis significantly impact the outcome of both diseases. The concurrent diseases resulted in lessening intestinal inflammation and fibrotic response. Further analysis revealed an alternation of the intestinal immunity and microbiome abundance in mice with both diseases compared to mono-diseased mice.

Similar to many previous studies using parasite antigens in acute colitis,^7^ ^,^ ^18^ ^,^ ^30^ our results found that natural infection of *S. mansoni* alleviated the symptoms and colonic histopathology in mice with chronic colitis (Fig. 1). But interestingly, the co-occurrence of both diseases also suppresses egg-induced fibrosis (Fig. 2). Because both diseases are immune-driven, we attempt to investigate the immune processes involved in these findings (Fig. 3). As expected, chronic schistosomiasis induced an increase in Th2 cytokines including IL-4, IL-5, and IL-10, which corroborates previous studies.^12^ In mice treated with chronic DSS, an increase of intestinal IL-4, IL-5, and IL-10 was noted, similar to what was previously reported.^31^ Finally, only IL-4 was slightly exacerbated in co-diseased mice, while IL-5 and IL-10 were decreased. Eosinophil recruitment has been proven during active inflammation in colitis.^32^ IL-4 and IL-5 are known for the production of IgE and eosinophils,^33^ which may take responsibility for intestinal inflammation. The two cytokines have also contributed to the development of schistosomiasis-associated fibrosis.^12^ However, the exacerbated IL-4 may also promote infiltration of anti-inflammatory macrophages^34^ ^,^ ^35^ and support the growth of regulatory T (Treg) cells,^36^ finally leading to the alleviation of inflammation and fibrosis. On the other hand, the decrease of IL-5 in SMDSS mice may reduce differentiation of eosinophils, thereby lowering the overall disease severity.^37^ Similarly, mice receiving anti-IL-5 exhibit reduced eosinophils infiltration and improved DSS-induced colitis symptoms.^38^ Fibrosis is also regulated by Treg cells, both positively and negatively.^39^ IL-10, although previously classified as a Th2 cytokine, is a major cytokine secreted by Treg cells that provides an immunomodulatory effect.^40^ Although increased IL-10 was found in IBD patients,^41^ the loss of IL-10 was also noted in some IBD patients.^42^ While SM mice showed an increase of IL-10, which could relate to the disease, the downregulation of IL-10 in SMDSS mice may thereby aid in protection. Another interesting question raised here is whether the viability of eggs will affect the occurrence of fibrosis. In the current study, we can only observe that all the eggs were intact [Fig. 2A, Supplementary data (Fig. 3A)] and assume that they were viable; however, the viability of the eggs strongly affects host-parasite interactions. For example, live eggs may secrete more soluble antigens than dead or unhealthy eggs to induce more immune cell infiltration and collagen deposition;^43^ therefore, whether DSS treatment or colitis itself may affect egg viability to reduce fibrosis may serve as a foundation for our future study.

Our study also investigated the immune response in the spleen, because both schistosomiasis and colitis are associated with the involvement of the splenic function.^20^ ^,^ ^44^ However, we obtained different results in the spleen compared to the colon. SM mice showed increased splenic expression of all the tested cytokines, but DSS mice showed only upregulation of *IL-2*. In SMDSS mice, *IL-4*, *IL-10*, and *IFN-γ* were downregulated. While IL-4 has contributed to hepatic-splenic schistosomiasis,^12^ ^,^ ^45^ the decrease in splenic *IL-4* expression may provide protection on the immune-driven pathology. In another study, low levels of IL-10 and IFN-γ were correlated to splenomegaly in *S. japonicum*-infected patients,^46^ similar to our finding showing an enlarged spleen in SMDSS mice (Fig. 1F). However, histological analysis revealed that splenomegaly observed in SMDSS mice was due to an enlarged white pulp, with increased immune cell activation [Supplementary data (Fig. 6A-C)]. Although the cell types were not differentiated in this study, we could postulate that the spleen was trying to activate more T or B cells to achieve the protective response in SMDSS mice, and this would serve as a basis for future study.

In addition, other cytokines such as IL-13 and transforming growth factor (TGF)-β may also be modulated in SMDSS mice, as they play an important role in fibrosis. It has been shown that IL-13 signalling induces the production of TGF-β in macrophages, leading to colitis-induced intestinal fibrosis.^47^ While schistosome eggs have also been characterised to induce IL-13 and TGF-β to lead to granuloma development,^12^ ^,^ ^48^ it is interesting that we observed a resolution of intestinal fibrosis in SMDSS mice. IL-1β is another cytokine coordinating the TGF-β signalling.^49^ IL-1β induces epithelial-mesenchymal transition dependent on the TGF-β signalling, leading to fibrosis. Our results show that intestinal IL-1β significantly decreased in SMDSS mice (Fig. 1M-N), possibly altering the IL-13 and TGF-β and thereby resulting in lower intestinal fibrosis. However, this hypothesis is not warranted based on the available evidence and may require future research.

Finally, the gut microbiome was analysed as it also plays a significant role in schistosomiasis and IBD^41^ ^,^ ^42^ and regulates the host immune response.^50^ While no difference was observed in alpha-diversity, significant differences were seen in beta-diversity (Fig. 5). Further analysis revealed several microbiomes that may be critical bacteria controlling the disease outcome in SMDSS mice. We identified an increased abundance of some probiotic bacteria such as *Lactobacillus*, *Closturia*_UCG-014, and *Peptococcaceae* family in SMDSS mice, where these bacteria have been shown to regulate immune response and limit inflammation.^51^ ^,^ ^52^ ^,^ ^53^ The abundance of the *Oscillospiraceae* family, which negatively influenced the metabolic status and health of the host,^54^ on the other hand, was increased in SM and DSS mice, and their abundance in SMDSS mice was returned to a similar level to control mice, possibly lead to the resolution of the disease. Our study also noted a significant decrease in the abundance of *Turicibacter* and *Erysipelotrichaceae* families in SM and DSS mice. Current knowledge is that *Turicibacter* and *Erysipelotrichaceae* regulate the host’s lipid metabolism and bile acids.^55^ ^,^ ^56^ ^,^ ^57^ Their abundance has also increased in probiotics-treated animals.^56^ While microbiome-controlled lipid metabolism highly regulates and limits intestinal inflammation,^58^ ^,^ ^59^ the loss of *Turicibacter* and *Erysipelotrichaceae* is therefore a possible cause of intestinal inflammation in SM and DSS mice. However, the abundance of the two bacteria remained at the same low level in SMDSS mice, suggesting that they may not be an essential bacterium in restricting the disease outcome.

Previously, a similar study where mice were acutely induced by DSS and infected with different sexes of SM also showed an altered microbiome composition and immune dysfunction.^60^ In that study, mice infected with only male worms were resistant to acute DSS-induced colitis, whereas mice infected with both sexes of worms developed a more severe DSS-induced colitis. This conflicts with our current study, where SM infection in mice ameliorated chronic DSS-induced colitis (Fig. 1). The differences between the outcomes may be due to the use of different models. In the former, acute colitis was induced by only seven days of DSS treatment,^60^ whereas in this study, chronic colitis was induced by three cycles of DSS treatment. Because withdrawing the DSS also withdraws colitis pathology,^61^ whether or not there is a co-occurrence of colitis and schistosome-associated injury may provide different outcomes of the disease. In addition, infection with only the male worms improved colitis symptoms,^60^ suggesting specific worm antigens, rather than egg antigens, may protect against the disease. Although the outcome between the acute^60^ and the chronic models showed different pathology outcomes, both models induced increased IL-4 and IL-10 levels (Fig. 3I, K), suggesting the same cytokine may have acted as a double-edged sword in colitis.

According to the above-discussed points, we know that the beneficial effects generated from this study are model-dependent. The animal model used in this study was simultaneously infected with SM and induced colitis. It is therefore questionable whether the same results can be obtained if colitis was induced before SM infection, or vice versa. It is known that IBD is usually accompanied by higher levels of Th1 cytokines.^62^ Because parasites are known as potent Th2 stimulators,^63^ parasite infection or its products achieve protection against colitis by reversing the cytokine imbalance.^10^ ^,^ ^17^ ^,^ ^60^ However, different life-cycle stages of schistosome induce entirely different kinds of immune responses; for instance, the Th1 response was invoked when cercariae penetrated and moved within the host, while the Th2 response dominated after the eggs were entrapped in the tissue. If colitis was induced before SM infection, where a strong Th1 response already dominates the body, a more vigorous Th1 response may be induced during cercarial infection. Therefore, future studies on different models may be required to provide foundational insights in this research field.

Parasite therapy has recently received total engrossment because of the proposed hygiene hypothesis. However, it is still uncertain whether parasitic treatment is safe, not to mention that it is still not approved as a treatment. Although previous study infecting Chron’s disease patients with *T. suis* have no observable adverse events,^10^ the possibility of complications such as malnutrition, gastrointestinal bleeding, and rectal prolapse must still not be overlooked, as *T. suis* has a global distribution and human infection do occur.^64^ ^,^ ^65^ Currently, parasite-related proteins are progressing well in treating autoimmune diseases as they pose no risk of infection;^66^ therefore, they may provide an alternative treatment for autoimmune diseases. Our results using the natural infection model here may provide another mechanism for how these parasite proteins alleviate autoimmune diseases.

In the current study, we identified that concomitant occurrence of chronic *S. mansoni* infection and chronic colitis aids protection against intestinal colitis and schistosome egg-induced intestinal fibrosis. Further investigation suggested that restoration of immune imbalance and dysbiosis may be the reason for this protection. Our results therefore represent an idea that schistosomiasis and chronic colitis may affect each other and provide a basis for future study using parasite therapy in IBD.
