# Supplementary material for: Concomitant occurrence of chronic Schistosoma mansoni infection and chronic colitis restore immune imbalance and dysbiosis leading to protection against intestinal colitis and schistosome egg-induced intestinal fibrosis
Source: Mem Inst Oswaldo Cruz. 2025 May 2;120:e240045. doi: 10.1590/0074-02760240045 (PMC12051921; doi:10.1590/0074-02760240045)
Supplement: Supplementary file 1 [file 1678-8060-mioc-120-e240045-s.pdf]

TABLE I

Scoring system for the disease activity index (DAI)

Score / Parameters	Percentage of weight loss	Presence of rectal bleeding	Stool consistency
0	0-1	No bleeding	Normal stool
1	2-5		
2	6-10	Occult blood*	Loose stool
3	11-20		
4	> 21	Macroscopic bleeding	Diarrheic stool

*Occult blood was analysed by the Guaiac method; the development of a blue colour is considered positive.

TABLE II

Primer pairs used in this study

Species	Gene name	Primer pairs (5' - 3')	GenBank accession
Mus musculus	*GAPDH*	Forward AGGTCGGTGTGAACGGATTTG<br>Reverse GGGGTCGTTGATGGCAACA	NM_008084
Mus musculus	*IL-1β*	Forward GAAATGCCACCTTTTGACAGTG<br>Reverse TGGATGCTCTCATCAGGACAG	NM_008361
Mus musculus	*COLA-1*	Forward GCTCCTCTTAGGGGCCACT<br>Reverse ATTGGGGACCCTTAGGCCAT	NM_007742
Mus musculus	*COLA-3*	Forward CTGTAACATGGAAACTGGGGAAA<br>Reverse CCATAGCTGAACTGAAAACCACC	NM_000090
Mus musculus	*IL-2*	Forward TGAGCAGGATGGAGAATTACAGG<br>Reverse GTCCAAGTTCATCTTCTAGGCAC	NM_008366
Mus musculus	*IL-4*	Forward CCCCAGCTAGTTGTCATCCTG<br>Reverse CAAGTGATTTTTGTCGCATCCG	NM_021283
Mus musculus	*IL-5*	Forward GCAATGAGACGATGAGGCTTC<br>Reverse GCCCCTGAAAGATTTCTCCAATG	NM_010558
Mus musculus	*IL-10*	Forward CTTACTGACTGGCATGAGGATCA<br>Reverse GCAGCTCTAGGAGCATGTGG	NM_010548
Mus musculus	*IFN-γ*	Forward GCCACGGCACAGTCATTGA<br>Reverse TGCTGATGGCCTGATTGTCTT	NM_008337
Mus musculus	*β-actin*	Forward GTGACGTTGACATCCGTAAAGA<br>Reverse GCCGGACTCATCGTACTCC	NM_007393
Mus musculus	*YWHAZ*	Forward TAGGTCATCGTGGAGGGTCG<br>Reverse GAAGCATTGGGGATCAAGAACTT	NM_001253806
Mus musculus	*18S*	Forward AGTTCCAGCACATTTTGCGAG<br>Reverse TCATCCTCCGTGAGTTCTCCA	NM_011296
Mus musculus	*HPRT*	Forward TCAGTCAACGGGGGACATAAA<br>Reverse GGGGCTGTACTGCTTAACCAG	NM_013556
Mus musculus	*SDHA*	Forward GGAACACTCCAAAAACAGACCT<br>Reverse CCACCACTGGGTATTGAGTAGAA	NM_023281
Mus musculus	*EEF2*	Forward CCGACTCCCTTGTGTGCAA<br>Reverse AGTTCAGGTCGTTCTCAGAGAG	NM_007907
Mus musculus	*TBP*	Forward CCTTGTACCCTTCACCAATGAC<br>Reverse ACAGCCAAGATTCACGGTAGA	NM_013684
Mus musculus	*B2M*	Forward TTCTGGTGCTTGTCTCACTGA<br>Reverse CAGTATGTTCGGCTTCCCATTC	NM_009735

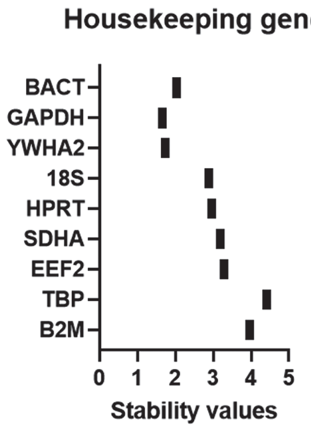

Gene name	Stability value
BACT	2.042
GAPDH	1.662
YWHA2	1.738
18S	2.898
HPRT	2.955
SDHA	3.195
EEF2	3.298
TBP	4.410
B2M	3.952

Fig. 1: stability score of different housekeeping genes. NormFinder analysis for a stable housekeeping gene among nine different candidate genes. A smaller stability value indicates a higher stability of the genes. The analysis revealed GAPDH as the most appropriate housekeeping gene in this study. BACT: beta-actin; GAPDH: glyceraldehyde-3-phosphate dehydrogenase; YWHAZ: tyrosine 3-monooxygenase/tryptophan 5-monooxygenase activation protein zeta; 18S: 18S ribosomal RNA; HPRT: hypoxanthine guanine phosphoribosyl transferase; SDHA: succinate dehydrogenase complex subunit A; EEF2: eukaryotic translation elongation factor 2; TBP: TATA-box binding protein; B2M: beta-2-microglobulin.

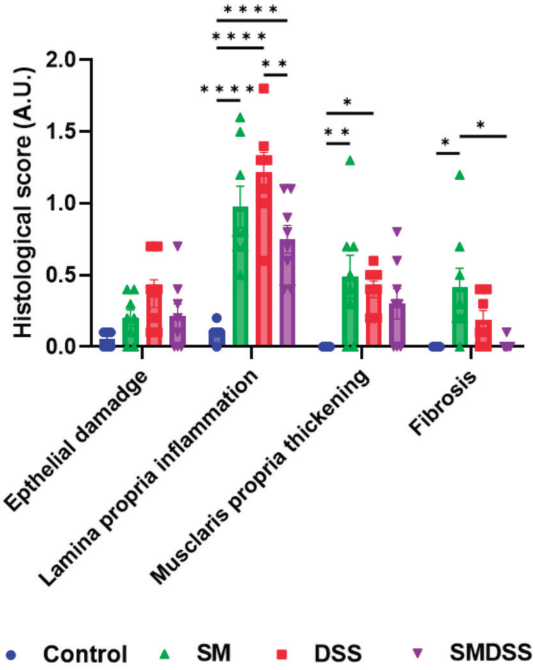

Fig. 2: different parameters of colon histology. (A) Different parameters of the colonic histology. Epithelial damage, lamina propria inflammation, muscularis propria thickening, and fibrosis were scored from 0 to 4: 0, absent; 1, mild; 2, moderate; 3, severe. The score from four criteria was summed to give a total histological score presented in Fig. 1H. $^{*}p < 0.05$; $^{**}p < 0.01$; and $^{****}p < 0.0001$. Significance determined by one-way analysis of variance (ANOVA).

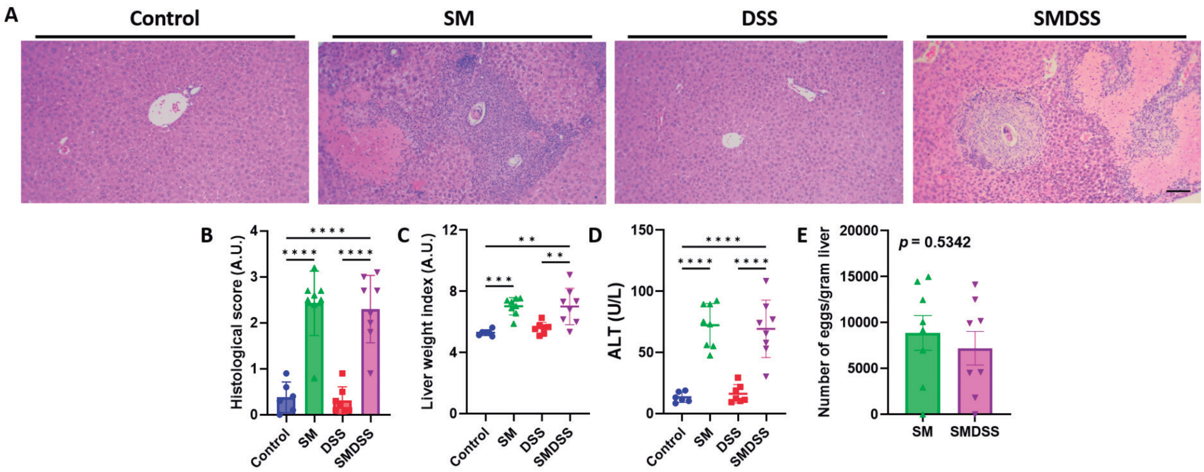

Fig. 3: concurrent schistosomiasis and colitis do not alter liver pathology. (A) Representative H&E-stained histological image of the liver. Scale bar represents 100 μm. (B) Histological score of the liver. (C) Liver weight index, calculated by the (liver weight divided by the body weight)×100. (D) Number of hepatic eggs. n = 6 mice in control group; n = 8 mice in SM and SMDSS group; and n = 5 mice in DSS group. Data are presented as mean ± SD. $^{**}p < 0.01$; $^{***}p < 0.001$; and $^{****}p < 0.0001$. (B and C) Significance determined by one-way analysis of variance (ANOVA). (D) Significance determined by t-test.

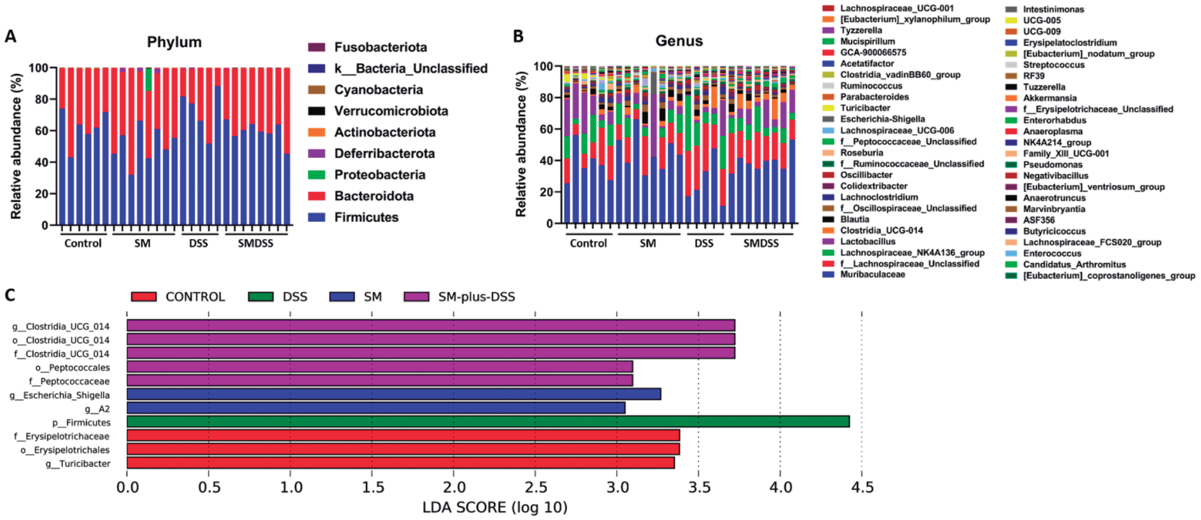

Fig. 4: analysis of gut microbiome in mice. (A-B) Individual taxa summary of bacterial phyla (A) and genera (B) from each group of mice, obtained by 16S rDNA sequencing. (C) Linear discriminant analysis (LDA) effect size (LEfSe) performed on the microbial community. Shown in the chart is taxa showing differences among the four groups.

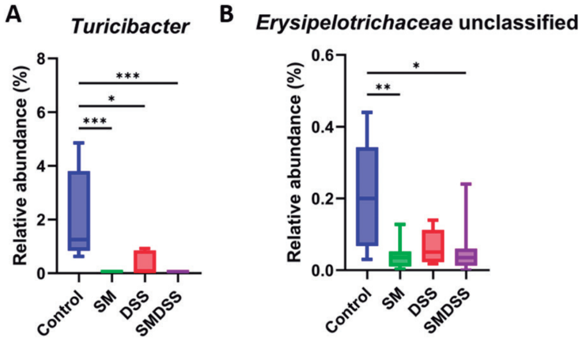

Fig. 5: abundance of *Turicibacter* and *Erysipelotrichaceae*. Relative abundance of (A) *Turicibacter* and (B) *Erysipelotrichaceae* family, obtained by 16S rDNA sequencing. Box and whisker plot display the median at the central line, 25-75 percentile at the box, and 10-90 percentile at the whiskers. *p < 0.05; **p < 0.01; and ***p < 0.001. Significance determined by one-way analysis of variance (ANOVA).

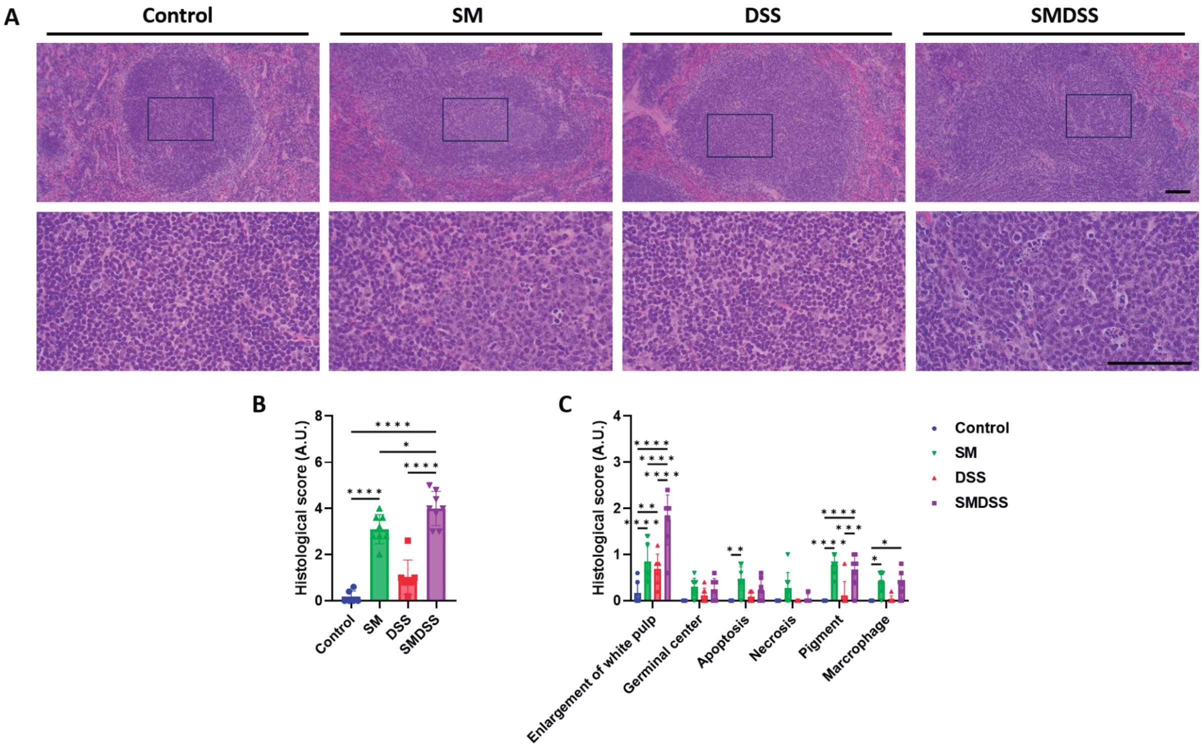

Fig. 6: histopathological analysis of the spleen. (A) Representative H&E-stained histological images of the spleen, showed at a 40× and 100× magnification. Scale bar represents 100 µm. (B) Total histological score of the spleen. (C) Different parameters of the splenic histology. Enlargement of white pulp was scored as: 0, absent; 1, mild; 2, moderate; and 3, pronounced. The presence of apoptosis, necrosis, pigments, and macrophages was each assigned a score of 0, absent and 1, present.
